# Correction: The Influence of Relatives on the Efficiency and Error Rate of Familial Searching

**DOI:** 10.1371/annotation/2544c2e3-0065-4d14-ab1c-3d3fcb0fee84

**Published:** 2014-01-06

**Authors:** Rori V. Rohlfs, Erin Murphy, Yun S. Song, Montgomery Slatkin

Figures 1 and 2 were incorrectly switched. The image currently appearing as Figure 1 belongs with the title and legend of Figure 2, and the image currently appearing as Figure 2 belongs with the title and legend of Figure 1. The titles and legends are in correct order. 

