# Supplementary material for: The Influence of Relatives on the Efficiency and Error Rate of Familial Searching
Source: PLoS One. 2013 Aug 14;8(8):e70495. doi: 10.1371/journal.pone.0070495 (PMC3743829; doi:10.1371/journal.pone.0070495)
Supplement: File S1 — Tables S1–5. (PDF) [file pone.0070495.s001.pdf]

Supporting Information File S1

The influence of relatives on the efficiency and error rate of familial searching

Rori V. Rohlfs[a], Erin Murphy[b], Yun S. Song[c,d], Montgomery Slatkin[a]

[a]Department of Integrative Biology, University of California, Berkeley, CA 94720, USA
[b]School of Law, New York University, New York, NY 10012, USA
[c]Computer Science Division, University of California, Berkeley, CA 94720, USA
[d]Department of Statistics, University of California, Berkeley, CA 94720, USA

Supporting Tables

Table S1: Normalized California census and prison population frequencies.

	simulated population				
	Asian	African American	European American	Latino American	Native American
Census population	.133	.0633	.410	.384	.0102
Prison population	.00626	.303	.268	.413	.00940

Note: We make use of these normative racial categorical constructs to estimate relevant population-specific identification rates. However, it's clear that membership in these categories is not mutually exclusive. By nature, assumptions must be made in the collection and tabulation of this sort of data. Further, as with all collected social data, some groups may be under or over-represented in data collection, and categorical results are subject to biases of reporting method (self-identified, inferred, etc.) (Spade and Rohlfs, *in review*).

Table S2: Projected percent of false parent-offspring leads which involve at least one individual from each population sample

		simulated population				
	total FPR	Vietnamese American	African American	European American	Latino American	Native American
Census population	5.73e-08	28.3	0.560	38.5	38.59	0.114
Prison population	3.84e-08	0.182	16.3	25.3	62.9	0.122

Table S3: Estimated percent of false sibling identifications which involve at least one individual from each population sample

		simulated population				
	total FPR	Vietnamese American	African American	European American	Latino American	Native American
Census population	3.01e-07	67.9	0.266	10.6	22.3	0.0849
Prison population	1.06e-07	0.459	15.2	13.5	73.1	0.199

Table S4: Parent-offspring test identification rates for different Y-sharing relatives and population samples where the set of reference Y-haplotypes was down-sampled to 103 haplotypes per population sample. The similarity of these results to those with the full set of Y-haplotypes indicates that the varying reference sample sizes are not driving population-based differences in identification rates.

	Vietnamese American	African American	European American	Latino American	Native American
parent-offspring	0.971109	0.917481	0.900600	0.904750	0.981429
sibling	0.260568	0.238175	0.247218	0.240125	0.346432
half-sib	0.052082	0.037330	0.039416	0.037430	0.099234
cousin	0.008184	0.004503	0.005204	0.004543	0.024529
half-cousin	0.002859	0.001183	0.001447	0.001275	0.010976
second cousin	0.001570	0.000522	0.000741	0.000616	0.007245

Table S5: Sibling test identification rates for different Y-sharing relatives and population samples where the set of reference Y-haplotypes was down-sampled to 103 haplotypes per population sample. The similarity of these results to those with the full set of Y-haplotypes indicates that the varying reference sample sizes are not driving population-based differences in identification rates.

	Vietnamese American	African American	European American	Latino American	Native American
sibling	0.786012	0.677311	0.636255	0.645179	0.831449
parent-offspring	0.750433	0.595312	0.521906	0.536782	0.815689
half-sib	0.166490	0.070316	0.055062	0.056729	0.240527
cousin	0.043701	0.010769	0.008384	0.008458	0.080298
half-cousin	0.016970	0.002642	0.002165	0.002175	0.038323
second cousin	0.009340	0.001130	0.000979	0.000928	0.024955
